# Effectiveness of speech therapy in treating vocal blocking tics in children with Tourette syndrome: Two case reports

**DOI:** 10.1177/13591045231177433

**Published:** 2023-05-24

**Authors:** Sini Peltokorpi, Auli Laiho, Vappu Carlson, Hanna Raaska

**Affiliations:** 1 Department of Psychology and Speech-Language Pathology, 8058 University of Turku, Turku, Finland; 2 Pediatric Neuropsychiatric Unit, 3836 HUS Helsinki University Hospital, Helsinki, Finland; 3 Voimavarakeskus Tempo Oy, Stuttering Resource Center Tempo, Espoo, Finland; 4 8054 The Social Insurance Institution of Finland, Helsinki, Finland

**Keywords:** Tourette syndrome, vocal blocking tics, blocking tics, blocking phenomena, complex tics, speech therapy, stuttering therapy

## Abstract

Tourette syndrome is characterized by at least two motor tics and one vocal tic, which persist for over a year. Infrequently, tics can manifest as blocking tics in speech when they prevent a person from starting to speak or interrupt their speech flow. Vocal blocking tics (VBTs) resemble stuttering, and they can be difficult to differentiate from each other. A previous report described two patients with severe VBTs who did not benefit from stuttering-therapy-based speech therapy and were treated effectively with cannabis-based medicine. Here, we present the cases of two patients, seven- and nine-year-old boys, who benefited from speech therapy in which stuttering therapy techniques were used. Detailed descriptions of the interventions are included. Further research is needed to test the effectiveness of speech therapy in treating VBTs in a larger group of children with Tourette syndrome.

## Introduction

A tic is a sudden, rapid, recurrent, and non-rhythmic motor movement or vocalization, which can be distinguished from other involuntary movements by its typical clinical characteristics. Tics are commonly sparse, but in some patients, they can be almost continuous. The frequency, intensity, number, complexity, and type of tics typically “wax and wane.” Tics can be situational, and they can be temporarily controlled. There is often a premonitory urge or tension preceding tics, which means a person’s ability to sense in advance that a tic is about to occur. The level of this urge varies across patients (Hartmann & Worbe, 2018; Szejko et al., 2022).

In Tourette syndrome (TS), there are at least two motor tics and one vocal tic, which are present over a one-year period (American Psychiatric Association, 2013). Its onset is typically before the age of 18. In the Diagnostic and Statistical Manual of Mental Disorders, Fifth Edition (5th ed.; *DSM-5;* American Psychiatric Association, 2013), TS belongs to the “neurodevelopmental disorders” group. The precise underlying mechanism is still unknown, but dysfunction of the cortico-basal ganglia neuronal networks is a widely accepted pathophysiological substrate in TS (Hartmann & Worbe, 2018). Comorbidities in TS are common. The most typical are obsessive-compulsive disorder (OCD), attention deficit/hyperactivity disorder (ADHD), mood and anxiety disorders, impulse control disorders, rage attacks, “impulsive” tic-like behaviors, and autism spectrum disorders (ASDs). These comorbidities often impact one's quality of life more significantly than the tics themselves (Hartmann & Worbe, 2018; Szejko et al., 2022). Although the official classification of TS is based on the presence of tics, it is a very heterogeneous condition. The variation and complexity of tics and the large number of comorbidities make personalized treatment approaches necessary.

In recent years, there has been increasing interest in psychosocial treatment interventions for tics, and in recent clinical guidelines, behavioral therapies are recommended as first-line interventions. An important base for behavioral therapy is the premonitory urge, as after learning to recognize this urge, tics can be suppressed. Behavioral therapies aim to enhance self-control and affect emotional or environmental factors. The habit reversal training/comprehensive behavioral intervention for tics (HRT/CBIT) has the strongest evidence for reducing tics. This manualized intervention consists of HRT, relaxation training, and a functional intervention to address situations that sustain or worsen tics (Andrén et al., 2022; Pringsheim et al., 2019b). In therapies based on exposure and response prevention (ERP), the aim is to increase urge tolerance and, in that way, to reduce tics. Tic suppression is practiced for prolonged periods of time, and exposure to the factors that induce them is increased (Andrén et al., 2022). Moreover, some medications, such as risperidone and aripiprazole, may have an effect in reducing the severity of tics (Pringsheim et al., 2019a).

The tics in TS can be simple or complex. Simple vocal tics consist of single sounds (e.g., sniffing), whereas complex vocal tics include more prominent tics, such as palilalia (repetition of the child’s own words or phrases) or echolalia (involuntary echoing of another person’s spoken words; Jimenez-Shahed, 2009). Infrequently, patients with TS have complex motor or vocal tics that interrupt voluntary action, leading to disfluency of speech or gait. These types of tics are called “blocking tics” or “blocking phenomena” (Ganos et al., 2015). Kaczyńska and Janik (2021) found that blocking tics can appear during speech, walking, running, and writing. At present, there is limited scientific knowledge about blocking tics.

When blocking tics occur during speech, they make the speech disfluent by blocking the initiation of speech or interrupting the speech flow. The result resembles stuttering, making their differential diagnosis difficult (Ganos et al., 2015; Jakubovski & Müller-Vahl, 2017; Kaczyńska & Janik, 2021). Kaczyńska and Janik (2021) provided an illustration of vocal blocking tics (VBTs) in the supplementary video attached to their paper (video 2), which shows how intrusive and disabling VBTs can be for the speaker. Although some children with TS have VBTs and stuttering-like disfluencies (blocks, prolongations, and repetitions) in speech, more typically, they have a high level of normal speech disfluencies (e.g., hesitations, interjections, and word repetitions; De Nil et al., 2005; Van Borsel et al., 2004; Van Borsel & Vanryckeghem, 2000).

In contrast to a high level of normal disfluencies typically observed in patients with TS, stuttering is characterized by repetitions and prolongations of sounds or syllables as well as blocks that may pause articulation, phonation, or breathing (American Psychiatric Association, 2013). Many speakers show secondary characteristics (e.g., increased muscle tension in the face or body, blinking of the eyes, turning of the eye and gazing away, and movements of the tongue, jaw, and head) and experience frustration, shame, and anxiety (Yaruss, 2007). This may lead to behavioral changes, such as the avoidance of difficult words, specific situations, or speaking in general (Lowe et al., 2017), and difficulties in participating in everyday life and social activities (Yaruss, 2007).

Most young children outgrow stuttering (Yairi & Ambrose, 2013), but in school-aged children, it is often persistent. Therefore, it is important to increase an acceptance of stuttering, build self-confidence, encourage communication and participation in social activities, and counsel family members and teachers. Many programs for school-aged children who stutter integrate techniques of speech restructuring (use of a novel speech pattern, such as slow articulation rate, to reduce or eliminate stuttering; see Onslow, 2022, p. 217) or stuttering modification (techniques to modify disfluent parts of speech; e.g., Van Riper, 1973) to cognitive-behavioral therapy, solution-focused therapy, and other methods that address the psychological effects of stuttering (e.g., Fourlas & Marousos, 2015; Murphy et al., 2007; Shields, 2018; Yaruss et al., 2012; see also Kelman & Nicholas, 2017; Yaruss et al., 2006). Stuttering modification treatment (Van Riper, 1973) begins with identifying the target behavior in detail and motivating the client. The therapy continues with desensitization to stuttering and the variations of stuttering. The actions in therapy include monitoring speech, analyzing the occurrence of stuttering, and modifying it either immediately after, during, or before the moment of stuttering. The treatment described by Van Riper (1973) resembles the CBIT treatment for tics (see e.g., Andrén et al., 2022).

Some children and youngsters with TS and speech disfluencies are treated with speech therapy (Jakubovski & Müller-Vahl, 2017; Van Borsel & Vanryckeghem, 2000). In some cases, phonic tics are treated by a speech and language therapist (SLT). Van Borsel and Vanryckeghem (2000) described an 18-year-old subject with TS whose disfluency in speech was first observed at the age of 14. He had difficulty formulating ideas, spoke in halting and rapid spurts, and slurred his articulation. Similarities were shown also in his handwriting, and all these characterize cluttering. At the age of 18, he received seven sessions of speech therapy, including exercises in slowed and syllable-timed speech and awareness training for bilabial tics. The speech therapy was effective in improving the patient’s speech fluency. Although the authors reported a slight increase in the frequency of phonic tics during conversation, these decreased while reading aloud after the speech therapy. The tics changed qualitatively to be less prominent.

To our knowledge, only one study has reported the effectiveness of speech therapy in treating VBTs in children with TS. Jakubovski and Müller-Vahl (2017) described two 19- and 16-year-old subjects with TS who had speech disfluencies caused by VBTs and palilalia. Their speech disfluencies were treated with speech therapy during their childhood and adolescence. The first subject underwent speech therapy over three years and the second subject over 10 years, without significant improvement in either case. The content of the speech therapy and the expertise of the SLTs were not clarified in the case report. Subsequently, the subjects were prescribed synthetic tetrahydrocannabinol, which was effective in improving their speech fluency.

In summary, previous studies have described the effectiveness of speech therapy in treating disfluencies in patients with TS, but it was ineffective in treating their VBTs. This is surprising as speech therapy for stuttering has many similarities with the behavioral therapies for tics. Here, we describe two patients with TS and VBTs who had positive outcomes with speech therapy. The children and their parents provided written informed consent to publish this report.

### Case presentation 1

#### History and initial presentation

The first patient (pseudonym Kevin) was referred to the Pediatric Neuropsychiatric Unit at the age of five years. During the evaluation, he was diagnosed with TS and ADHD. As a part of the treatment process, he and his parents received psychoeducation about tics and TS, but it was not focused on VBTs. He was prescribed atomoxetine for ADHD and risperidone (0.5 mg) for tics. He had no cognitive delay. At the time of the SLT assessment, Kevin was seven years and four months old. He had had occupational therapy for one year but no prior behavioral therapy for tics or appointments with an SLT. The parents indicated that Kevin had had the VBTs since he was 3–4 years old, and they came in one-month cycles. The intensity of the VBTs varied. Kevin was highly aware of them and experienced premonitory urges of the most severe tics.

#### Assessment

During the SLT assessment, Kevin was cooperative, and it was easy to communicate with him. He had several motor tics during the appointment: stiffening of torso, opening of mouth, blinking of eyes, and head movements towards the elbow. However, he had no VBTs. Kevin’s speech comprehension and production skills were not evaluated with linguistic tests because his language skills corresponded to his age level as assessed by a neuropsychologist. Kevin’s speech was fluent in naming tasks and tasks requiring repetition. He had good narrative skills and pronounced all the Finnish sounds correctly. He did not have problems in oral motor functioning or diadochokinesis (the ability to make fast, repeated, and alternate speech movements, which is often evaluated in assessing speech motor skills; Staróbole Juste et al., 2012).

The parents provided videos that showed Kevin’s VBTs. These made Kevin’s speech very disfluent and difficult to understand by disrupting his speech in the beginning and middle of sentences. During the VBTs, Kevin’s breathing was blocked; he also had abnormal facial expressions, opened his mouth several times, blinked his eyes, and made coughing-like sounds. At times, he touched his throat while trying to speak. During the most difficult moments, he had no control over his speech and was on the verge of tears. The parents reported that sometimes it took over a minute for Kevin to finish a sentence.

#### Goals and treatment

Kevin’s therapy was conducted by the second author, an SLT with two decades of experience specializing in the treatment of stuttering, who is also a psychotherapist. The goal in Kevin’s speech therapy was to improve his fluency of speech by learning techniques he could use to modify his speech. Moreover, the aim was to strengthen his resilience. There were 21 individual therapy sessions and one meeting with parents and the staff members of the Pediatric Neuropsychiatric Unit. The therapy sessions were conducted in the clinic once per week for seven months. One of the parents participated in each session approximately half of the time. The parents practiced with Kevin between the sessions at home and helped him to generalize his skills in everyday situations. Kevin was highly motivated in the treatment of his VBTs. At the start of the therapy, he asked for help in speaking because he had no control over the VBTs, and he felt like he was suffocating.

Kevin’s speech therapy included elements of psychoeducation. Throughout the therapy, his experiences in speaking and his feelings related to the VBTs were openly discussed. An important focus of the therapy was also to support Kevin’s willingness to communicate and not allow the VBTs to restrict his participation in social activities. The importance of sharing his thoughts and interacting with others despite the VBTs was emphasized.

An essential part of Kevin’s speech therapy was practicing techniques to control the VBTs. First, his normal speech production was analyzed. Kevin was interested in exploring how the body produces speech and in drawing the “speech machine,” which is the anatomy of organs and muscles needed for speech. Then, it was identified what happens in VBTs, since it is important to be aware of the behavior and the difference between fluent speech and VBTs.

In the early stage of treatment, the focus was on respiration and speaking during exhalation. Kevin was advised to gently start his airflow before beginning phonation. Focusing on airflow prevented the blocking of his vocal cords. He also practiced monitoring and modifying both his speech rate and muscle tension during speech to gain control over the increased muscle tension he experienced during his VBTs. Kevin also practiced pausing and phrasing speech, refraining from producing long sentences during one exhalation in order to maintain optimal airflow during speech. With these techniques, applied and combined with the speech restructuring and stuttering modification therapies (e.g., Onslow, 2022, p. 217), Kevin learned quickly to speak without VBTs in the therapy. Next, attention was paid to identifying and modifying his residual tics. Kevin started to notice when a residual and milder VBT occurred and its premonitory urge. This high level of awareness and use of the techniques made it possible for him to finish sentences without VBTs.

After four therapy sessions, the VBTs did not reappear in Kevin’s speech in a typically varying pattern, and he experienced a period of 10 weeks without them. After that, the VBTs returned for a few days, but disappeared again. During the remaining months of the therapy, he had some residual VBTs. Since Kevin had learned ways to control his VBTs, however, their duration decreased, and he experienced less frustration and anxiety. Thus, the VBTs were no longer as disturbing as before, although his parents reported that they were more prevalent when Kevin was tired or upset. However, even in these situations, he was now able to talk and finish his sentences. Since his condition had considerably improved, the dose of risperidone was reduced, without a negative impact on the VBTs.

#### Outcomes of the therapy

After his speech therapy, Kevin has been followed up by the Pediatric Neuropsychiatric Unit. He still has other types of tics, but the VBTs no longer prevent him from speaking, and he has control over them. Kevin’s speech therapy was aimed specifically at modifying the VBTs, and the results have lasted for more than two years after the end of his therapy.

### Case presentation 2

#### History and initial presentation

The second patient (pseudonym Mike) was diagnosed with TS and ASD (Asperger’s syndrome) at the age of eight. He had no cognitive delay. For his tics, he was prescribed aripiprazole (7.5–10 mg/day). He has had weekly multidisciplinary family-based neuropsychiatric rehabilitation sessions for one and a half years, as well as 11 physiotherapy sessions. Mike has not received behavioral therapy for his tics, nor has he received any psychoeducation. At the time of the SLT assessment, Mike was nine years and four months old. He suffered from motor blocking tics affecting his speech and gait, which led to a sudden cessation of speaking and walking. His VBTs resulted in difficulties with speech initiation, interruption of speech, and problems completing sentences. They came in two-to three-month cycles and were highly disturbing, as he was not able to speak fluently. The VBTs also caused tension, severe headache, and pain in his jaw and neck. Mike was ashamed of his tics and tried to inhibit them. Moreover, he was bullied at school because of his TS. Mike’s mother asked for help for ease the pain and speech disfluencies caused by his VBTs.

#### Assessment

The SLT assessment consisted of an interview with Mike’s mother and an online meeting with Mike. During the first 30 minutes, Mike was verbally fluent, used grammatically correct utterances, and expressed himself with well-formulated ideas. His articulation was correct. At times, he cleared his throat and opened his jaw wide but was nevertheless able to complete sentences. Near the end of the meeting, Mike started to turn his head away from the screen and cover his face with his hands in an effort to hide his tics.

#### Goals and treatment

Mike was first prescribed 20 sessions of speech therapy, and three guidance sessions were provided to his parents. The goals were to improve Mike’s knowledge of the techniques that he can use to modify his speech and to foster his openness and acceptance of VBTs and TS. Therapy was provided by the same SLT as with Kevin. After the 20 sessions, the therapy continued with 10 more sessions, two school meetings, and five guidance sessions for the parents.

The therapy sessions took place in video meetings and were scheduled once per week during the first six months, and after that, every other week. Mike wanted to be alone with the SLT, so typically, the therapist and his mother conversed after Mike’s session. This worked very well and gave Mike the ability to control the intensity of interaction by sometimes stepping away from the screen.

The first step in Mike’s therapy was to build a therapeutic relationship in which he would feel accepted. In the early stages of the therapy (sessions 1−10), Mike expressed hatred towards his VBTs, denied them, and refused many suggested activities. Around the 10th session, however, he finally felt comfortable enough to participate actively in the therapy. Mike’s therapy sessions were carefully structured in advance, and his mother prepared him for the content of the next session two days beforehand. When Mike knew what to expect, it was easier for him to participate.

Mike’s speech therapy included elements of psychoeducation. The differences between people were explored, and his personal strengths and capacities were identified. This helped Mike to understand that even when he feels that his VBTs are unacceptable, he has other traits and skills of which he can be proud. Mike expressed concerns about how trying to control the VBTs might worsen them. His reactions were analyzed with respect to having no control over the VBTs (“I can’t speak, I can’t control the VBTs at all.”) or too much control over them (“It’s stressful, the VBTs get worse, and I get angry and frustrated.”). At school, the VBTs caused him embarrassment, and he feared others’ negative reactions. During sessions 20−35, it was discussed with Mike’s teacher how to make speaking easier for him at school.

Different techniques were practiced for controlling his VBTs from the 11th session onward. He still did not experience a premonitory urge before a VBT. Variations in speaking were tested (e.g., rapidly/slowly, tensed/relaxed manner, with disruptions/continuity) to give Mike a sense of the possibility of modifying his speech. A mind map including Mike’s ideas of different ways to deal with VBTs was created (e.g., relaxing the mind and preventing stress, intentionally pausing and not rushing in speech, talking while exhaling, focusing on the continuity of speech movements, and doing the opposite of VBTs), and all these ideas were practiced.

Mike’s ability to monitor fluency failures was first practiced by his monitoring of the SLT’s speech, and then eventually his speech (a technique applied from the stuttering modification approach; Van Riper, 1973). Gradually, Mike became more aware of his VBTs and learned how to notice a premonitory urge. This made it possible for him to take pauses in his speech and let the VBTs occur between the words. Mike found it very useful to use the analogy of traffic lights while he was speaking; he learned to pause during a red light between sentences. This was applied from the speech restructuring treatments for stuttering (e.g., Kelman & Nicholas, 2017). The pause was a time for Mike to let the VBTs come out, and after the pause, he could speak the next sentences without disruptions in speech flow. This technique was useful for him, in combination with premonitory awareness, which gave him a signal to pause at the right time. Previously, his VBTs had occurred in the middle of words and sentences, so that sometimes it was difficult for the listener to understand Mike’s speech. His VBTs did not disappear altogether, but they no longer disturbed his speech flow and communication to the same extent. Mike developed some control over his VBTs and was able to time them to occur in suitable places during his speech.

In therapy and at home, Mike was making progress, but he still experienced major difficulties at school. He felt that his behavior worsened because he was not understood at school. Mike behaved aggressively and impulsively, and he also expressed suicidal thoughts, reflecting the major impact of TS on his life. These stressful factors may have exacerbated Mike’s VBTs. Even though they still occurred during controlled pauses (“traffic lights”) and not within words, Mike reported that these controlled pauses were necessarily long since the duration of the VBTs was also long. At this point, Mike started to communicate partially through writing. Eventually, he was diagnosed with ADD and was prescribed guanfacine. With suitable medication, Mike’s VBTs became milder, and he gained control over them again. The combination of medication and techniques to control his speech behavior helped Mike communicate more easily.

#### Outcomes of the therapy

The main outcome of Mike’s therapy was the change in his attitude and a move from denial towards acceptance of his TS. He is now more open and willing to talk about TS and VBTs and finds it useful to tell others about his condition. It was only possible to work directly with his VBTs after major attitudinal changes. Mike learned the valuable techniques of timing his VBTs between sentences so that they did not occur within words and to monitor the VBTs in advance. These techniques, combined with suitable medication, helped him to communicate with others so that his speech was understandable for listeners.

## Discussion

The two patients presented in this report benefited from speech therapy for their VBTs. In Kevin’s case, a rather short therapy was sufficient to arm him with techniques that he could use successfully to modify his speech when the VBTs occurred. This reduced his stress and resentment towards the VBTs and made it possible for him to participate more actively in conversations. The improvement in Kevin’s condition could not be attributed to the natural wax-and-wane cycle of tics, as his ability to control his speech during the VBTs was gained from the techniques used in the therapy, and this ability has been maintained for over two years since the end of his therapy.

In Mike’s case, the main outcomes were related to his attitude towards the VBTs. Although he learned techniques that helped him to monitor his VBTs and modify his behavior, this was not possible before he was able to accept the VBTs as part of him. Therefore, the therapy process took longer than with Kevin. Neither Kevin nor Mike had similar outcomes with their earlier treatments, including medication, other therapies, and psychoeducation. It remains unanswered whether CBIT or ERP could have provided similar benefits to these patients as speech therapy because neither of them received behavioral interventions for their VBTs.

The speech therapy was based on the principles of stuttering therapy (see e.g., Laiho et al., 2022; Onslow, 2022, p. 217), and the positive outcomes of the therapies they received are therefore attributable to the knowledge about the treatment methods for stuttering. However, the speech therapy provided was not manualized; it was individualized for both children, and it integrated elements of several stuttering therapy methods. Our results are in contrast with the results of Jakubovski and Müller-Vahl (2017). They suggested that patients with TS may not benefit from stuttering-based speech therapy, which was the case in the two young men with TS in their case report. Moreover, they suggested that patients with TS and VBTs can be misdiagnosed as having a speech pathology disorder. Making the right diagnosis is, of course, vitally important. However, from our perspective, the relationship between VBTs and speech-language pathology should be re-evaluated, since despite the underlying diagnoses, VBTs are a communication disorder causing severe impairment in speech fluency.

Discussion around the acceptance and awareness of tics is an important part of the treatment of children with TS. Psychoeducation played a major role in Mike’s therapy and his improved well-being. In Kevin’s case, psychoeducation was included in the therapy, but the emphasis was on practicing the techniques to control his VBTs. Hence, his positive treatment outcomes cannot be explained by psychoeducation. The findings therefore underline the importance of individual planning and implementation of psychoeducation for children with VBTs. This is crucial as psychoeducation is recommended as the first part of the treatment for children with TS (Andrén et al., 2022). However, Andrén et al. (2022) mention that there is a lack of evidence about which elements should be addressed in psychoeducation. It would be ideal if the professionals providing psychoeducation to children with VBTs had knowledge on speech production, speech fluency, and VBTs to be able to share information about them and analyze speech together with children. In this respect, SLTs have significant knowledge based on their education. Moreover, it is important to discuss children’s speech-related experiences and feelings and strengthen their psychological resilience. These components are all naturally part of many stuttering therapy methods (e.g., Fourlas & Marousos, 2015; Kelman & Nicholas, 2017; Van Riper, 1973; Yaruss et al., 2012).

It is unlikely that psychoeducation alone is sufficient for treating VBTs in children with TS, as they represent a complex form of tics. Both CBIT and ERP are evidence-based interventions for treating tics. However, there are no evidence-based indications as to which intervention works the best for whom (Andrén et al., 2022). Thus, children with different types of tics may benefit from different interventions. Moreover, the interventions have different impacts on speaking in persons with VBTs. When ERP is used, the child needs to suppress the VBTs for prolonged periods of time. This means that the child is not able to speak until the urge to tic is over. Therefore, using ERP may be challenging as it prevents persons with VBTs from expressing themselves. In CBIT and stuttering therapy, the focus is on modifying the target behavior. Where CBIT aims to apply incompatible responses with VBTs, stuttering therapy aims to directly modify the stuttering/VBT by using the knowledge of how to speak with control. The latter may have some advantages as the strategies are applied directly to speaking instead of a competing response. Therefore, we suggest that stuttering therapy be included in future studies that compare the effectiveness of interventions for children with VBTs.

TS that includes VBTs is a complex condition. Since the appearance and quality of VBTs may vary, it is essential not only to learn to control them but also to build problem-solving skills so that the speaker can adjust the techniques in case of future variations in VBTs. This is well recognized also in the treatment of stuttering. Therapy for VBTs can be started by providing support for the acceptance of tics even before the speaker experiences the premonitory urge. When people with TS can openly talk about their VBTs, it is possible to analyze them, which in turn helps to develop better awareness of the premonitory urges.

There are several limitations to this report. As the report does not have a design for a case study, it is missing measurement data, such as Yale Global Tic Severity Scale (YGTSS; Leckman et al., 1989) or Premonitory Urge for Tics Scale (PUTS; Woods et al., 2005) and focuses on retrospectively reporting on the treatment for VBTs. Moreover, as only two children were included in this report, and both were treated in an individualized way, the results do not apply to all children with VBTs.

For the well-being of children with TS, the ability to communicate their needs and emotional states is essential. Learning how to control their VBTs may reduce the distress they experience with this condition and lead to further positive outcomes.
